# Responsiveness of electrical nociceptive detection thresholds to capsaicin (8 %)-induced changes in nociceptive processing

**DOI:** 10.1007/s00221-016-4655-z

**Published:** 2016-05-03

**Authors:** Robert J. Doll, Guido van Amerongen, Justin L. Hay, Geert J. Groeneveld, Peter H. Veltink, Jan R. Buitenweg

**Affiliations:** 1 Biomedical Signals and Systems, MIRA Institute for Biomedical Technology and Technical Medicine, University of Twente, Zuidhorst, ZH-222, Drienerlolaan 5, PO BOX 217, Enschede, The Netherlands; 2 Centre for Human Drug Research, Leiden, The Netherlands

**Keywords:** Nociception, Intra-epidermal electrical stimulation, Capsaicin, Psychophysics

## Abstract

Pain disorders can be initiated and maintained by malfunctioning of one or several mechanisms underlying the nociceptive function. Psychophysical procedures allow the estimation of nociceptive detection thresholds using intra-epidermal electrical stimuli. By varying the temporal properties of electrical stimuli, various contributions of nociceptive processes to stimulus processing can be observed. To observe the responsiveness of nociceptive thresholds to changes in nociceptive function, a model of capsaicin-induced nerve defunctionalization was used. Its effect on nociceptive detections thresholds was investigated over a period of 84 days. A cutaneous capsaicin (8 %) patch was applied for 60 min to the upper leg of eight healthy human participants. Single- and double-pulse electrical stimuli were presented in a pseudo-random order using an intra-epidermal electrode. Stimuli and corresponding responses were recorded on both treated and untreated skin areas prior to capsaicin application and on days 2, 7, 28, and 84. Increases in electrical detection thresholds at the capsaicin area were observed on days 2 and 7 for single-pulse stimuli. Detection thresholds corresponding to double-pulse stimuli were increased on days 7 and 28, suggesting a delayed and longer lasting effect on double-pulse stimuli. In the present study, it was demonstrated that the responsiveness of detection thresholds to capsaicin application depends on the temporal properties of electrical stimuli. The observation of capsaicin-induced changes by estimation of detection thresholds revealed different time patterns of contributions of peripheral and central mechanisms to stimulus processing.

## Introduction

Chronic pain disorders can be initiated and maintained by malfunctioning of one or several mechanisms underlying the nociceptive function (Mendell 2011; Sandkühler 2009; Woolf 2011). Quantification of the contributions of these mechanisms could help identifying malfunctioning at a peripheral and central level. Although several methodologies exist to quantify pain processing, such as psychophysical and neurophysiological assessment of sensory function, it remains difficult to detect specific malfunctioning mechanisms. This could hamper mechanism-based treatment of (potential) pain syndromes such as small fiber neuropathy (Devigili et al. 2008), complex regional pain syndrome (Borchers and Gershwin 2014), or persistent post-surgical pain (Kehlet et al. 2006). Therefore, there is a need for methodology for improved observation of nociceptive processing.

Quantitative sensory testing (QST) methods allow psychophysical assessments of sensory function (Arendt-Nielsen and Yarnitsky 2009). These methods include application of a broad range of stimulus types such as thermal, mechanical, or electrical and recording corresponding responses. Particularly, thermal and electrical stimuli can be used for preferential stimulation of nociceptive nerve fibers (Inui and Kakigi 2012; Kodaira et al. 2014; Mouraux et al. 2010). An advantage of electrical stimulation is the accurate control of stimulation timing, allowing well-defined stimuli with temporal resolutions in the order of tens of µs. Varying the temporal properties of rectangular-wave stimuli, such as the pulse-width (PW), number of pulses (NoP), and inter-pulse interval (IPI), allows probing of phenomena such as the strength–duration relationship (Rollman 1969; Weiss 1901) or temporal summation of post-synaptic activity (Mouraux et al. 2014; van der Heide et al. 2009). Observation of these phenomena is relevant, especially when changes in nociceptive function are to be identified. For example, peripheral changes are expected in patients with small fiber neuropathy (e.g., neuronal swelling or nerve defunctionalization), and central changes during central sensitization. Probing changes in nociceptive function requires a method which allows the simultaneous observation of responses to stimuli with varying stimulus parameters.

Within a single experiment, participants can be presented with a mixed sequence of stimuli with various predefined temporal properties. The simultaneous recording of responses to these stimuli and estimation of corresponding psychophysical curves could help observing the mechanisms involved in nociceptive processing in more detail. The feasibility of this method was demonstrated in a 10-min experiment including healthy human participants (Doll et al. 2016). Stimuli with different temporal properties were presented in a pseudo-random order, and psychophysical curves were estimated per stimulus type. Differences in curves between single-pulse stimuli with varying PWs were related to the strength–duration curve and reflected peripheral nociceptive processing. The difference between the curves of a single-pulse stimulus and a double-pulse stimulus demonstrated a facilitatory effect present in double-pulse stimuli.

Observation of pharmacologically induced changes in nociceptive function in healthy human participants is a next step in identifying the usability of intra-epidermal electrical stimulation. A good candidate for inducing temporary changes is the application of a cutaneous patch containing an 8 %-dose of capsaicin. It was shown that the application of capsaicin results in temporary nerve defunctionalization by retraction of Aδ and C fibers (Anand and Bley 2011; Kennedy et al. 2010; Polydefkis et al. 2004). Skin biopsies show that the intra-epidermal nerve fiber density (IENFD) is reduced after capsaicin application and shows a return to baseline within 6 months (Kennedy et al. 2010; Polydefkis et al. 2004). As a result, nociceptive, but also tactile, thresholds are temporarily increased for up to a week after application (Kennedy et al. 2010; Mouraux et al. 2010; Ragé et al. 2010). Moreover, temporary sensitization occurs at both a peripheral and central level right after capsaicin application (Sandkühler 2009; Woolf 2011).

In this study, a single application of an 8 %-dose capsaicin patch was used to induce changes in nociceptive function in healthy human participants. The main objective was to simultaneously observe the responsiveness of multiple nociceptive thresholds to changes in nociceptive function over a time period of 84 days after capsaicin application. The nociceptive function was psychophysically probed in a simple detection task using intra-epidermal electrical stimulation with a variety in temporal electrical stimulus properties. Series of stimulus–response pairs were recorded prior to capsaicin application and on days 2, 7, 28, and 84 on both treated and untreated skin areas.

## Methods

### Participants

After approval of the LUMC Ethics Committee and in accordance with the declaration of Helsinki, 12 healthy participants (six men, six women) were enrolled after providing written informed consent. For logistic reasons, four participants could not take part in this study, leaving eight participants (five men, three women; mean age = 22.5, SD = 2.0). Inclusion criteria were: 18–65 years old and a body mass index between 18 and 30 kg m^−2^, good medical condition defined as absence of clinically significant findings in their medical history, physical examination and vital signs. Exclusion criteria were pregnancy, illicit drug use, frequent caffeine use (>8 units/day), smokers (>5 cigarettes/day), extreme responders to capsaicin 0.075 % cream, skin abnormalities and abnormal ECG or blood pressure. Moreover, in a separate part of the study (not reported here), erythema or reddening of the skin on the upper back was measured. As this cannot be measured in dark toned skin, participants with dark toned skin (Fitzpatrick skin type V or VI) were excluded from the study. No over-the-counter medication within 3 days of nociceptive measurements was allowed. During the study, participants were asked to refrain from strenuous physical exercise, use of all (methyl)xanthenes, and alcohol. Female participants attended study-day 0 while in the follicular phase.

### Experiment design

Participants visited the laboratory on 5 days during a period of 84 days. Two adjacent areas were marked on participants’ distal lateral thigh non-dominant leg using transparencies. A cutaneous 6 × 6 cm patch containing 8 % w/w (640 µg/cm^2^) capsaicin (Qutenza, Astellas Pharma B.V., Leiden, the Netherlands) was applied about 10 cm proximal to the knee for 60 min. The adjacent untreated area was about 9 cm proximal to the capsaicin treated area and served as the control. A 60 min pre-treatment with EMLA 5 % was applied before capsaicin application. Psychophysical recordings took place prior to capsaicin application (D0), and on days 2, 7, 28, and 84 on both the treated and untreated areas. For 9 min, participants were presented electrical stimuli and corresponding responses were recorded (i.e., detected or undetected).

### Test-stimuli

**Fig. 1:**
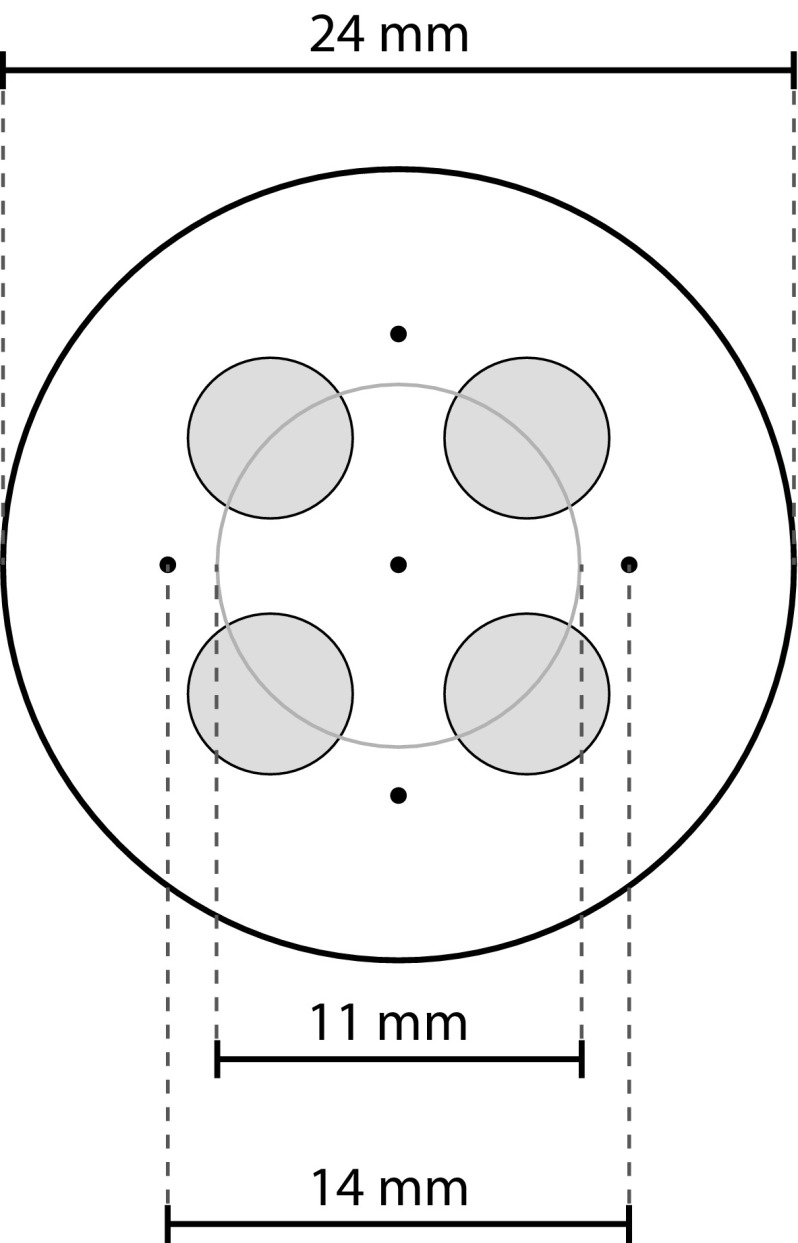
Schematic representation of the needle electrode. The electrode consists of four interconnected 5-mm diameter disk electrodes and five interconnected needle electrodes

An electrode consisting of an array of five interconnected needles and four interconnected flat electrodes with a diameter of 5 mm was attached to either the treated or untreated skin area (see Fig. 1 for a schematic representation of the electrode and see (Steenbergen et al. 2012) for more details). The needles served as cathode and a conducting pad covering the flat electrodes as anode (Steenbergen et al. 2012). The needles protruded 0.5 mm from the electrode surface. Each visit, the skin area to which the electrode was attached first was randomly determined. The computer controlled constant current stimulator was developed at our group and is similar to the one used by (Roosink et al. 2011; Steenbergen et al. 2012; van der Heide et al. 2009).

Cathodic rectangular pulses were applied as test-stimuli using four different combinations of temporal properties (Table 1). Stimulus amplitudes were selected according to an adaptive probing procedure (Doll et al. 2014). The procedure started with a pre-defined set of five equidistant stimulus amplitudes between 0 and 0.4 mA for single-pulse stimuli and between 0 and 0.2 mA for double-pulse stimuli. The amplitude of the upcoming stimulus was randomly selected from this set. All amplitudes in the set were increased and decrease with a fixed step size after a not-detected stimulus and detected stimulus, respectively. The step size was 0.1 mA for single-pulse stimuli and 0.05 mA for double-pulse stimuli. The different stimulus settings were presented in a randomly intermingled sequence.

**Table 1:** Temporal stimulus properties: pulse-width (PW), number of pulses (NoP), and inter-pulse interval (IPI)

	PW (µs)	NoP	IPI (ms)
Setting 1 (P1_PW210)	210	1	–
Setting 2 (P1_PW525)	525	1	–
Setting 3 (P2_PW525_IPI20)	525	2	20
Setting 4 (P2_PW525_IPI50)	525	2	50

During the experiment procedure, participants were instructed to press and hold a response button until a stimulus was detected. While undetected, the stimulator continued to apply stimuli with an inter-stimulus interval ranging between 2.5 and 2.9 s. After a stimulus was detected, participants were to release the button and to press the button again after about a second. Therefore, the inter-stimulus interval after a detected stimulus was increased with about a second, resulting in an average inter-stimulus interval of about 3.7 s. A custom computer program (written in LabVIEW 2011, SP1) controlled all stimulation procedures, as well as the registration of stimulus amplitudes in mA, stimulation times in milliseconds, and responses to stimuli (i.e., detected or not-detected).

### Statistical analysis

All data preparation was performed in MATLAB 8.1 (MathWorks, Inc, Natick, MA, USA). Statistical modeling was performed using the lme4 library (Bates et al. 2014) in the R software package (R Core Team 2014). Generalized linear mixed models (GLMM) using a logit link function were built to estimate the detection probability given the stimulus amplitude. Type III Wald *χ*^2^ statistics were used to test the main and interaction effects of the fixed effects. Confidence intervals of the regression parameters were based on Wald-*z* statistics. Threshold estimates were obtained from the regression parameters, and corresponding standard errors were approximated using the Delta procedure (Faraggi et al. 2003; Moscatelli et al. 2012). Post-hoc comparisons were performed using Bonferroni *p* value correction.

#### Effect of temporal stimulus properties

A GLMM was built to study the effect of stimulus properties (i.e., setting) on the detection probability in terms of thresholds and slopes. Only the D0 data obtained at the untreated skin area were included. The intercept, stimulus amplitude (mA), setting, stimulation time (s), and the interaction between the stimulus amplitude and setting were included as fixed effects. Between-subjects random effects were included for the intercept, stimulus amplitude, and setting. An unstructured covariance matrix was used to model the random effects. To speed up the estimation process, the stimulation time variable was centered and scaled prior to the analysis (*z*-transform). Detection thresholds and slopes were compared between all settings.

#### Effect of capsaicin

For each set of temporal stimulus properties (i.e., setting), a GLMM was built to study the effect of capsaicin on the detection threshold. The intercept, stimulus amplitude, study day, location, stimulation time, and the interaction between study day and location were included as fixed effects. Between-subjects random effects were included for the intercept, stimulus amplitude, study day, and location. An unstructured covariance matrix was used to model the random effects. To speed up the estimation process, the stimulation time variable was centered and scaled (*z*-transform) prior to the analysis. The thresholds on each stimulation location were compared on each study day.

Initially, a model including all data was tried to fit which would allow to study differential effects of capsaicin on the detection probability of all sets of temporal stimulus properties in more detail than presented here. However, due to the sparse amount of data and complexity of the regression model (mainly due to a triple interaction effect in the fixed effects part and multiple interaction effects in the random effects part), the model was poorly fit as convergence could not be reached. Therefore, it was decided to fit a separate model for each set of temporal stimulus properties. Future studies including more data could try to fit a more complex model, allowing to study the differential effect in more detail.

## Results

All eight participants completed the experiment. Out of a total of 14,857 stimuli, 843 stimuli were excluded for analysis due to technical issues. About 46 stimuli and corresponding responses (mean = 46.4, SD = 3.3) were available per participant, per study day, per skin area, per setting. Therefore, participants were presented with approximately 1840 stimuli in total.

### Effect of temporal stimulus parameters

Table 2 presents the results of the GLMM analyses. Only the intercept and the interaction between the stimulus amplitude and set of temporal stimulus properties (i.e., setting) significantly affected the detection probability. The stimulus amplitude, setting, and stimulation time did not affect the detection probability.

**Table 2:** Comparison between temporal stimulus properties: type III Wald statistics

Parameter	*χ* ^2^ (*df*)	*p*
(Intercept)	12.1 (1)	<0.001
Stimulus amplitude	3.3 (1)	0.070
Setting	6.5 (3)	0.090
Time	1.7 (1)	0.186
Stimulus amplitude × setting	53.2 (3)	<0.001

**Fig. 2:**
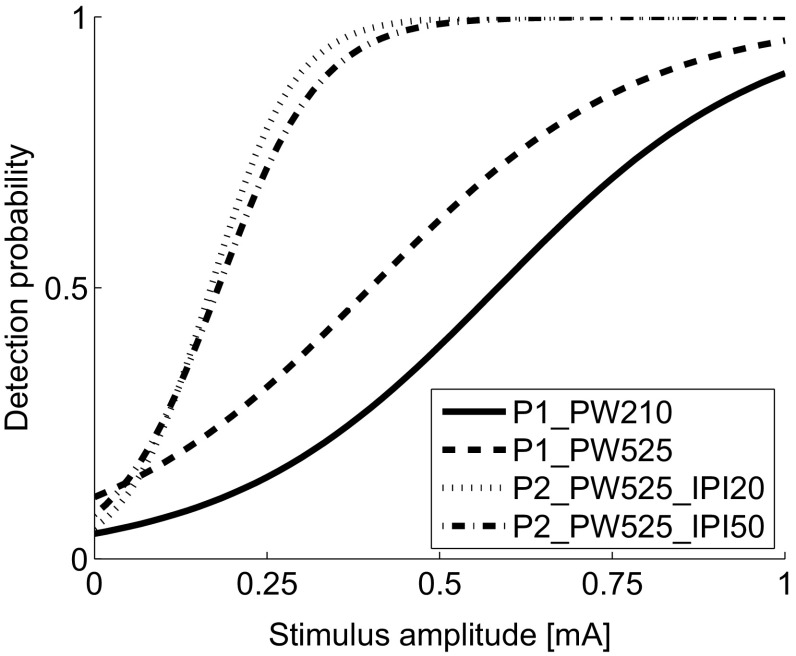
Psychophysical curves for each combination of temporal stimulus properties on the untreated skin area prior to capsaicin application (Table 1). The *curves* are obtained from the regression parameters (Table 3)

The estimated log-odds and corresponding 95 % confidence intervals are presented in Table 3. Note that the stimulation time variable was *z*-transformed prior to the analysis. As the mean stimulation time was about 4.5 min, the obtained parameters can be interpreted as the expected value at a stimulation time of 4.5 min, and thus at the middle of the experiment. The regression parameters are inverse-logit transformed to obtain the psychophysical curves for all settings (Fig. 2).

**Table 3:** Comparison between temporal stimulus properties: regression parameter estimates of the fixed effects and corresponding confidence intervals

Parameter	Estimate (SE)	95 % CI
(Intercept)	−3.02 (0.87)	[−4.71, −1.32]
Stimulus amplitude	5.17 (2.85)	[−0.41, 10.75]
Setting		
Setting 2	0.97 (0.54)	[−0.10, 2.03]
Setting 3	0.22 (0.86)	[−1.46, 1.89]
Setting 4	0.60 (0.84)	[−1.06, 2.25]
Stimulation time	−0.10 (0.08)	[−0.26, 0.05]
Stimulus amplitude × setting		
Amplitude × setting 2	−0.04 (0.68)	[−1.36, 1.29]
Amplitude × setting 3	11.43 (1.71)	[8.09, 14.77]
Amplitude × setting 4	8.32 (1.59)	[5.20, 11.44]

Presented values are the log-odds. See Table 1 for details on the settings

**Fig. 3:**
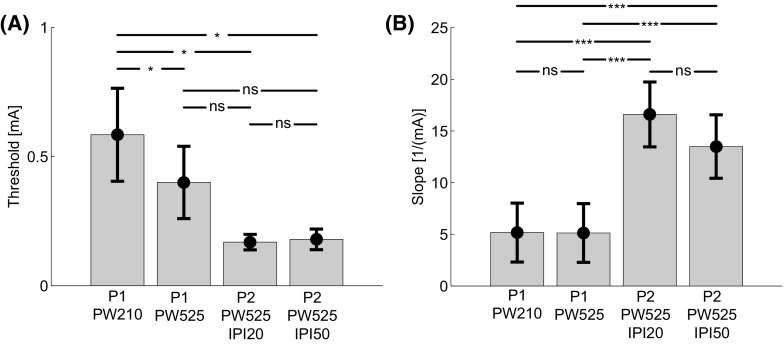
Estimated thresholds (**a**) and slopes (**b**) and corresponding standard errors for each combination of temporal stimulus properties (Table 1). *, **, and *** indicate a significant mean difference with a value of *p* < 0.05, *p* < 0.01, and *p* < 0.001, respectively

The estimated thresholds and slopes are presented in Fig. 3. Post-hoc comparisons between settings showed that a decrease in threshold was observed when increasing the PW from 210 to 525 µs, and when increasing the PW and NoP from a single 210 µs pulse to a double 525 µs pulse. No differences were observed when comparing the threshold for the single 525 µs pulse stimulus with the double 525 µs pulse stimulus. Differences between slopes were observed when increasing the NoP, but not when increasing the PW or IPI.

### Effect of capsaicin

Table 4 presents the results of the GLMM analyses. For all four combinations of stimulus properties (Table 1), the intercept, stimulus amplitude, stimulation time, and the interaction between study day and location significantly affected the detection probability. Moreover, the detection probability was not affected by study day and location in all four settings.

**Table 4:** Effect of capsaicin: type III Wald statistics

Parameter	Setting 1		Setting 2		Setting 3		Setting 4	
	*χ* ^2^ (*df*)	*p*	*χ* ^2^ (*df*)	*p*	*χ* ^2^ (*df*)	*p*	*χ* ^2^ (*df*)	*p*
(Intercept)	26.8 (1)	<0.001	38.1 (1)	<0.001	56.9 (1)	<0.001	42.8 (1)	<0.001
Stimulus amplitude	20.3 (1)	<0.001	34.2 (1)	<0.001	45.5 (1)	<0.001	30.3 (1)	<0.001
Study day	3.0 (4)	0.565	7.8 (4)	0.101	6.5 (4)	0.162	0.6 (4)	0.965
Location	0.4 (1)	0.551	2.3 (1)	0.132	0.8 (1)	0.380	0.8 (1)	0.364
Stimulation time	10.4 (1)	0.001	39.1 (1)	<0.001	36.1 (1)	<0.001	23.1 (1)	<0.001
Day × location	47.4 (4)	<0.001	72.8 (4)	<0.001	64.5 (4)	<0.001	69.6 (4)	<0.001

See Table 1 for details on the settings

**Fig. 4:**
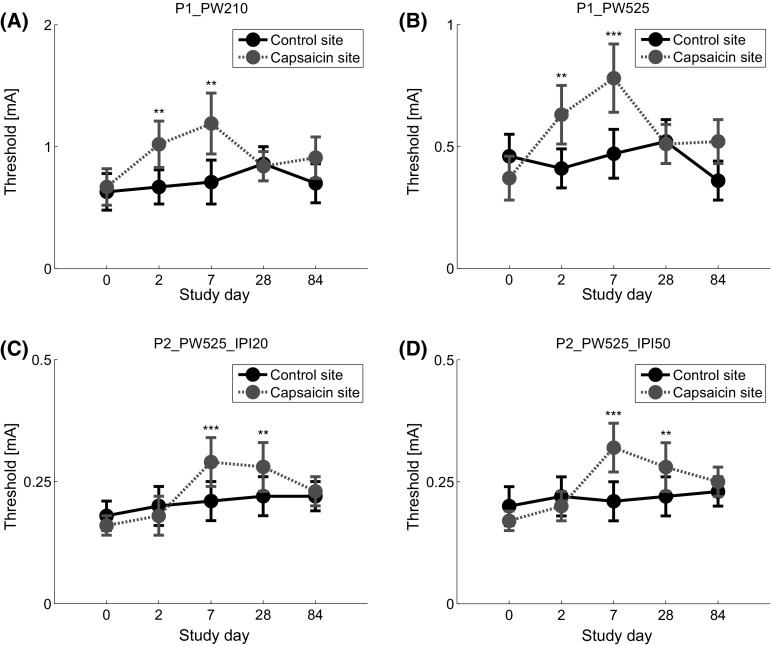
Estimated thresholds and corresponding standard errors for all four combinations of temporal stimulus properties (Table 1). *, **, and *** indicate a significant mean difference between the thresholds obtained at the capsaicin treated skin area and untreated area with a value of *p* < 0.05, *p* < 0.01, and *p* < 0.001, respectively. Note the difference in *y* axis in the four subfigures

The estimated log-odds for the regression parameters and corresponding 95 % confidence intervals are presented in Table 5. Note that the stimulation time variable was *z*-transformed prior to the analysis. As the mean stimulation time was about 4.5 min, the obtained parameters can be interpreted as the expected value at a stimulation time of 4.5 min, and thus at the middle of the experiment. The estimated thresholds and corresponding standard errors of all settings and days are presented in Fig. 4. When comparing the thresholds between skin areas on the same study day, an increase in threshold was observed for single-pulse stimuli (i.e., setting 1 and setting 2) on days 2 and 7. An increase in threshold was observed on days 7 and 28 for double-pulse stimuli.

**Table 5:** Effect of capsaicin: regression parameter estimates of the fixed effects and corresponding confidence intervals

	Setting 1		Setting 2		Setting 3		Setting 4	
	Estimate (SE)	95 % CI	Estimate (SE)	95 % CI	Estimate (SE)	95 % CI	Estimate (SE)	95 % CI
(Intercept)	−2.15 (0.42)	[−2.97, −1.34]	−2.39 (0.39)	[−3.14, −1.63]	−2.88 (0.38)	[−3.62, −2.13]	−2.71 (0.41)	[−3.52, −1.90]
Stimulus amplitude	3.41 (0.76)	[1.93, 4.90]	5.21 (0.89)	[3.46, 6.95]	16.28 (2.41)	[11.55, 21.01]	13.89 (2.52)	[8.95, 18.84]
Study day								
Day 2	−0.14 (0.49)	[−1.10, 0.83]	0.27 (0.56)	[−0.82, 1.37]	−0.44 (0.60)	[−1.61, 0.73]	−0.30 (0.51)	[−1.30, 0.69]
Day 7	−0.28 (0.35)	[−0.96, 0.40]	−0.08 (0.30)	[−0.66, 0.50]	−0.51 (0.46)	[−1.42, 0.40]	−0.21 (0.44)	[−1.07, 0.66]
Day 28	−0.77 (0.61)	[−1.97, 0.44]	−0.31 (0.47)	[−1.24, 0.62]	−0.64 (0.41)	[−1.45, 0.16]	−0.29 (0.45)	[−1.18, 0.60]
Day 84	−0.25 (0.49)	[−1.21, 0.72]	0.51 (0.46)	[−0.39, 1.40]	−0.71 (0.59)	[−1.86, 0.44]	−0.42 (0.61)	[−1.62, 0.78]
Location								
Capsaicin	−0.14 (0.24)	[−0.61, 0.32]	0.45 (0.30)	[−0.14, 1.04]	0.28 (0.32)	[−0.34, 0.90]	0.29 (0.32)	[−0.34, 0.91]
Stimulation time	−0.16 (0.05)	[−0.26, −0.06]	−0.30 (0.05)	[−0.39, −0.20]	−0.28 (0.05)	[−0.38, −0.19]	−0.23 (0.05)	[−0.32, −0.13]
Study day × location								
Day 2 × capsaicin	−1.04 (0.27)	[−1.58, −0.51]	−1.60 (0.28)	[−2.14, −1.06]	0.05 (0.25)	[−0.45, 0.55]	−0.03 (0.25)	[−0.53, 0.46]
Day 7 × capsaicin	−1.50 (0.29)	[−2.07, −0.93]	−2.06 (0.28)	[−2.61, −1.52]	−1.65 (0.26)	[−2.16, −1.13]	−1.84 (0.26)	[−2.36, −1.32]
Day 28 × capsaicin	0.20 (0.26)	[−0.31, 0.72]	−0.42 (0.26)	[−0.94, 0.09]	−1.25 (0.27)	[−1.77, −0.73]	−1.21 (0.27)	[−1.74, −0.67]
Day 84 × capsaicin	−0.57 (0.28)	[−1.11, −0.03]	−1.25 (0.28)	[−1.79, −0.71]	−0.47 (0.26)	[−0.98, 0.04]	−0.61 (0.26)	[−1.12, −0.11]

Presented values are the log-odds. See Table 1 for details on the settings

## Discussion

In this study, a single application of an 8 % dose capsaicin patch was used to induce changes in nociceptive function in healthy human participants. The main objective was to simultaneously observe the responsiveness of multiple nociceptive thresholds to changes in nociceptive function over a time period of 84 days after capsaicin application. Nociceptive function was psychophysically probed in a simple detection task using intra-epidermal electrical stimulation with a variety in temporal electrical stimulus properties. Series of stimulus–response pairs were recorded prior to capsaicin application and on days 2, 7, 28, and 84 on both treated and untreated skin areas.

A needle electrode was used for intra-epidermal electrical stimulation. Recent studies have shown that this type of stimulation device allows the preferential stimulation of nociceptive Aδ fibers, provided that the stimulus amplitudes are below twice the detection threshold, this method allowed preferential stimulation of Aδ fibers (Legrain and Mouraux 2013; Mouraux et al. 2010). In the present experiment, stimulus amplitudes were chosen according to an adaptive stimulus selection procedure (Doll et al. 2014; 2015) such that the amplitudes were always near the detection threshold. Therefore, the contributions of tactile Aβ fibers to the threshold are negligible (see (Doll et al. 2016) for a comprehensive explanation).

Throughout the experiment, stimuli with four different temporal properties (Table 1) were presented to participants in a pseudo-random order. The parameter values were experimentally chosen, but keeping two phenomena in mind: the strength–duration relationship for nociceptive fibers, and temporal summation. The PWs were chosen near the expected chronaxie value for nociceptive fibers. Thresholds recorded for this value are also likely to be most sensitive to peripheral changes. IPI values were chosen longer than 5 ms to avoid stimulating during the refractory period of nerve fibers and relatively short such that both pulses are not perceived individually. Moreover, these values are also near the expected time constants of temporal summation of postsynaptic potentials. The differences between the PW, NoP, and IPI of these stimuli allowed simultaneous observation of various contributions of nociceptive processes to stimulus processing (Doll et al. 2016). Differences in detection probabilities of stimuli with varying PWs provide information about strength–duration properties. Differences caused by an increase in the NoP (e.g., increasing the NoP from a single-pulse stimulus to a double-pulse stimulus) may inform about peripheral and/or central facilitation or inhibition. The IPI then, while effects are relatively small (Doll et al. 2016), might provide information regarding the time constants of facilitation or habituation.

For the comparison between the detection probability of a single-pulse (NoP = 1) and double-pulse stimulus (NoP = 2), it should be noted that the detection probability of a double-pulse stimulus, *p*_d_, depends on the detection probabilities of each of the individual pulses, *p*_*s*1_ and *p*_*s*2_, according to probability summation: *p*_d_ = 1 − (1 − *p*_s1_)(1 − *p*_s2_). If the separate detection probabilities of both pulses are independent and equal, i.e., *p*_s1_ = *p*_s2_, we refer to this as to pure probability summation, i.e., *p*_d,pure_ = 1 − (1 − *p*_s1_)^2^. Based on pure probability summation, the detection threshold of a double-pulse stimulus is equal to the amplitude resulting in a 0.29 detection probability of a single stimulus. When, at a certain stimulation amplitude, the observed detection probability of a double-pulse stimulus is lower or higher than expected by pure probability summation (i.e., *p*_d_ < *p*_d,pure_ or *p*_d_ > *p*_d,pure_), this indicates that the detection of the second pulse is inhibited or facilitated, respectively, by the presence of the first pulse. This effect was ascribed to either peripheral or central facilitation (Doll et al. 2016). Peripheral facilitation might be induced due to subthreshold superexcitable properties of fibers (Bostock et al. 2005) and central facilitation due to temporal summation of post-synaptic potentials or short term plasticity (Zucker and Regehr 2002).

### Effect of temporal stimulus properties

When considering only the data obtained on the control location on day 0 (i.e., prior to capsaicin application), a decrease in threshold was observed when increasing the PW of a single-pulse stimulus from 210 to 525 µs (Fig. 3a). This effect of the PW is governed by the strength–duration curve (Geddes 2004; Rollman 1969) reflecting peripheral mechanisms of nociceptive processing and is similar to previous findings (Doll et al. 2016).

Although a tendency toward a lower threshold for double-pulse stimuli than for single-pulse stimuli with the same PW (Fig. 3a), no significant difference was observed. The slope, however, was significantly steeper for double-pulse stimuli than for single-pulse stimuli (Fig. 3b). Moreover, no difference in threshold and slope was observed for double-pulse stimuli with different IPI values. In previous studies, a difference between thresholds of single and double-pulse stimuli was observed (Doll et al. 2016). Moreover, it was also demonstrated that the detection probability of the second pulse was facilitated. In that study, however, more participants were included and more SRPs were available for the estimation process. Therefore, we conclude that future studies should include more data if the effect of temporal stimulus properties on both the threshold and slope is of interest.

No differences in thresholds and slopes were observed for double-pulse stimuli when the IPI value was increased from 20 to 50 ms. In a previous study, it was already demonstrated that IPI has a relatively small effect on the detection probability. Only a small change in threshold was observed when increasing the IPI value from 10 to 100 ms. As mentioned above, more participants and SRPs were available for the estimation of the detection probability. Additionally, the range of IPI values was broader than the range used in the present study. Therefore, the IPI range should be increased, as well as the number of SRPs when the effect of IPI is of interest.

### Effect of capsaicin

The two adjacent stimulation locations were close together, possibly affecting the quality of the control recordings. However, the detection thresholds on the control location remained relatively constant over the study period, regardless of the combination of temporal stimulus properties (Tables 4, 5). Moreover, as the distance between the capsaicin patch and control location was relatively large (>2 cm), capsaicin diffusion toward the control skin area is unlikely (Selim et al. 2010). Therefore, it is unlikely that the capsaicin diffused into the control skin area and induced peripheral or central changes. Additional measures could also be considered to study, for example, the presence of secondary hyperalgesia at the control site.

The thresholds recorded on the treated location were affected by the capsaicin application and showed increases lasting for several days. The time profiles of single-pulse thresholds with different PWs were similar: thresholds were increased on days 2 and 7 and returned to baseline value within 28 days. The time profiles of double-pulse stimuli with varying IPI values were also similar, but different than the profile of single-pulse stimuli: thresholds were increased on days 7 and 28, and returned to baseline value within 84 days. The different time profiles of single and double-pulse thresholds suggest that various nociceptive processes are affected by capsaicin application and that these changes might be observable in psychophysical thresholds. Possible explanations for the differences in time patterns are discussed in the paragraphs below.

Studies have shown a reduction in IENFD within a week with similar capsaicin patches (Kennedy et al. 2010; Knolle et al. 2013; Malmberg et al. 2004). It is likely that the IENFD was already reduced on day 2 as studies using a lower capsaicin dose found reductions within 2 days (Polydefkis et al. 2004). The reduction in IENFD indicates a retraction of nerve fibers (O’Neill et al. 2012) and thereby increasing the distance between the electrode surface and nerve fibers. As a result, higher stimulation currents are required to reach and activate the retracted nerve fibers. Therefore, an increase in detection thresholds can be expected when the IENFD is reduced and likely explains the increase in threshold for single-pulse stimuli.

As the detection thresholds for single-pulse stimuli were increased on day 2, an increase in threshold was expected for double-pulse stimuli as well. Based on pure probability summation, the expected detection threshold for double-pulse stimuli is equal to the amplitude resulting in a detection probability of 0.29 for a single-pulse stimulus. With an increase in single-pulse threshold, a slight decrease in slope could be expected as well, resulting in a less effective increase in double-pulse threshold. However, the thresholds for double-pulse stimuli were not increased on day 2. This suggests an increased facilitation on the detection probability of the second pulse in comparison with day 0. This increased facilitation was no longer observed on day 7, as an increase in thresholds for double-pulse stimuli were observed on that day. Whether the lower threshold for double-pulse stimuli can be explained by increased peripheral activity due to, for example, increased subthreshold superexcitability (Bostock et al. 2005), increased central activity [e.g., due to increased temporal summation of post-synaptic potentials or short term plasticity (Zucker and Regehr 2002)] or a combination of the two is unclear at this point. Further research is required to be able to distinguish peripheral from central contributions. For example, similar to methods as described by (Bostock et al. 2005) and (Burke et al. 2009) could be used to study subthreshold superexcitability in cutaneous nociceptive fibers.

While the detection thresholds for single-pulse stimuli returned to baseline value within 28 days, the thresholds for double-pulse stimuli were still increased on day 28. As the detection probability of the first pulse of a double-pulse stimulus is equal to the detection probability of a single-pulse stimulus, the detection probability of the second pulse must be inhibited or less facilitated. Again, this inhibition or decreased facilitation could be caused by peripheral mechanisms, by central mechanisms, or by a combination of both. Estimating slopes of psychophysical curves per study day and per stimulation location could aid in deciding whether inhibition or decreased facilitation occurs on day 28. However, due to variability, we were unable to obtain reliable slope estimates and are therefore unable to verify a change in slope. As estimation of the threshold is relatively simple in contrast to estimation of the slope (King-Smith and Rose 1997; Kontsevich and Tyler 1999), future studies focussing on the slope could either increase the number of stimulus–response pairs, or include more participants. These studies preferably also take into account possible effects of the stimulus selection procedure used in this experiment on the estimation quality of the slope.

## Conclusion and outlook

In the present study, it was demonstrated that the responsiveness of detection thresholds to capsaicin-induced changes in nociceptive processing depends on the temporal properties of electrical stimuli. The detection thresholds to single-pulse stimuli were increased on days 2 and 7 after capsaicin application, while the detection thresholds to double-pulse stimuli were increased on days 7 and 28. Overall, we demonstrated that the use of intra-epidermal electrical stimulation can be used to explore changes in nociceptive processing. A better understanding of nociceptive processing in healthy controls can be achieved by computational models based on the underlying neurophysiology. A next step for further exploration of intra-epidermal stimulation as a method to observe contributions of nociceptive mechanisms to stimulus processing is to incorporate the methods presented in this paper in a clinical setting. A first group of patients could include those scheduled for surgery. The incidence of persisting pain development after surgery is high (Perkins and Kehlet 2000), while treatment of settled persistent pain is relatively ineffective (Apfelbaum et al. 2003). Following nociceptive changes prior and post-surgery using intra-epidermal electrical stimulation could be of additional value to existing QST measures used for clinical purposes (Backonja et al. 2013) in describing the state of the nociceptive system.
